# A Good Clinician

**Published:** 1914-10-24

**Authors:** 


### "A Good Clinician."

PRESENTATION TO SIR ROBERT SIMON.

SIR ROBERT SIMON, who has resigned the position of senior honorary physician at the Birmingham General Hospital after thirty-five years' service, was publicly thanked by the Lord Mayor and other prominent citizens last week for the attention he has given to the sick poor of the city during his long association with the institution. The Lord Mayor, Alderman W. H. Bowater, presided over a large audience comprised of subscribers to the special presentation, an event which took place in the Board Room of the hospital. The Chairman of the Board, Mr. J. R. H. Smyth, asked the Lord Mayor to accept a portrait of Sir Robert, painted by Mr. Edward S. Harper, which represents him nearly full face, seated and wearing the gown of a doctor of medicine, to be hung in the Board Room of the hospital. Sir John Holder, Bart., as chairman of the house committee, then presented to Lady Simon a diamond tiara, which was, he said, an acknowledgment of the high appreciation of the devoted services Sir Robert Simon had rendered to the General Hospital, and by his work on the Samaritan Committee. An album containing an illuminated resolution and list of subscribers was presented to Sir Robert by Mr. Gilbert Barling, senior surgeon, who said that the highest praise he felt he could give to Sir Robert was that he was a good clinician; he had a certain gift in the recognition and treatment of a disease, and if a man had not that gift, in his opinion, he never made a good physician nor a good surgeon.

